# Functional improvement of dystrophic muscle by repression of utrophin: let-7c interaction

**DOI:** 10.1371/journal.pone.0182676

**Published:** 2017-10-18

**Authors:** Manoj K. Mishra, Emanuele Loro, Kasturi Sengupta, Steve D. Wilton, Tejvir S. Khurana

**Affiliations:** 1 Department of Physiology and Pennsylvania Muscle Institute, Perelman School of Medicine, University of Pennsylvania, Philadelphia, Pennsylvania, United States of America; 2 Perron Institute for Neurological and Translational Science, University of Western Australia, Perth, Australia; 3 Centre for Comparative Genomics, Murdoch University, Perth, Australia; Rutgers University Newark, UNITED STATES

## Abstract

Duchenne muscular dystrophy (DMD) is a fatal genetic disease caused by an absence of the 427kD muscle-specific dystrophin isoform. Utrophin is the autosomal homolog of dystrophin and when overexpressed, can compensate for the absence of dystrophin and rescue the dystrophic phenotype of the *mdx* mouse model of DMD. Utrophin is subject to miRNA mediated repression by several miRNAs including let-7c. Inhibition of utrophin: let-7c interaction is predicted to 'repress the repression' and increase utrophin expression. We developed and tested the ability of an oligonucleotide, composed of 2'-O-methyl modified bases on a phosphorothioate backbone, to anneal to the utrophin 3'UTR and prevent let-7c miRNA binding, thereby upregulating utrophin expression and improving the dystrophic phenotype *in vivo*. Suppression of utrophin: let-7c interaction using bi-weekly intraperitoneal injections of let7 site blocking oligonucleotides (SBOs) for 1 month in the *mdx* mouse model for DMD, led to increased utrophin expression along with improved muscle histology, decreased fibrosis and increased specific force. The functional improvement of dystrophic muscle achieved using let7-SBOs suggests a novel utrophin upregulation-based therapeutic strategy for DMD.

## Introduction

Duchenne Muscular Dystrophy (DMD) is the most common fatal X-linked neuromuscular disease. DMD is caused by mutations in the dystrophin gene that lead to quantitative and qualitative disturbances in the expression of the dystrophin protein [1–3]. Dystrophin is member of the spectrin superfamily of proteins that includes the spectrins, alpha-actinins and utrophin [4, 5]. Dystrophin provides structural integrity to muscle by linking the actin cytoskeleton to the extracellular matrix *via* the muscle membrane bound dystrophin-glycoprotein complex (DGC) [6, 7]. Lack of functional dystrophin disrupts this link, resulting in severe and progressive muscle weakness and wasting. While great progress has been made in understanding the etiology and pathogenesis of DMD, the disease remains incurable and is the focus of concerted global efforts toward developing therapies [3, 8]. Utrophin is considered the autosomal homologue of dystrophin as it has extensive sequence similarity with dystrophin [9–12]. Functionally, utrophin shares a number of important structural motifs with dystrophin, such as the N-terminal, central spectrin repeat rod domain, cystine rich region and the C-terminal tail as well as functional properties such as F-actin binding and ability to associate with the DGC [7, 9]. Utrophin is similar but not identical to dystrophin and differences such as nNOS [13] and microtubule binding potential have also been reported [14]. Additionally, differences exist both in the tissue and subcellular distribution [10, 15, 16]. Importantly from a therapeutic point of view, multiple studies have demonstrated that utrophin upregulation using a variety of different means (e.g. transgenic, viral vectors, pharmacological) can functionally rescue the dystrophic phenotype in the *mdx* mouse model of DMD [17–21]. Additionally, even extremely high levels of utrophin (e.g. using transgenic means) have not been associated with toxicity [3, 22], making utrophin upregulation an attractive therapeutic strategy for DMD. Indeed, a number of utrophin promoter trans-activator molecules have been identified and are in various states of preclinical and clinical development [3, 18, 23, 24]. However, utrophin is also subject to repression by several miRNAs including let-7c [25, 26]. Inhibiting the utrophin: let-7c interaction offers a potential method to upregulate utrophin expression for DMD gene therapy [26].

**Fig 1:**
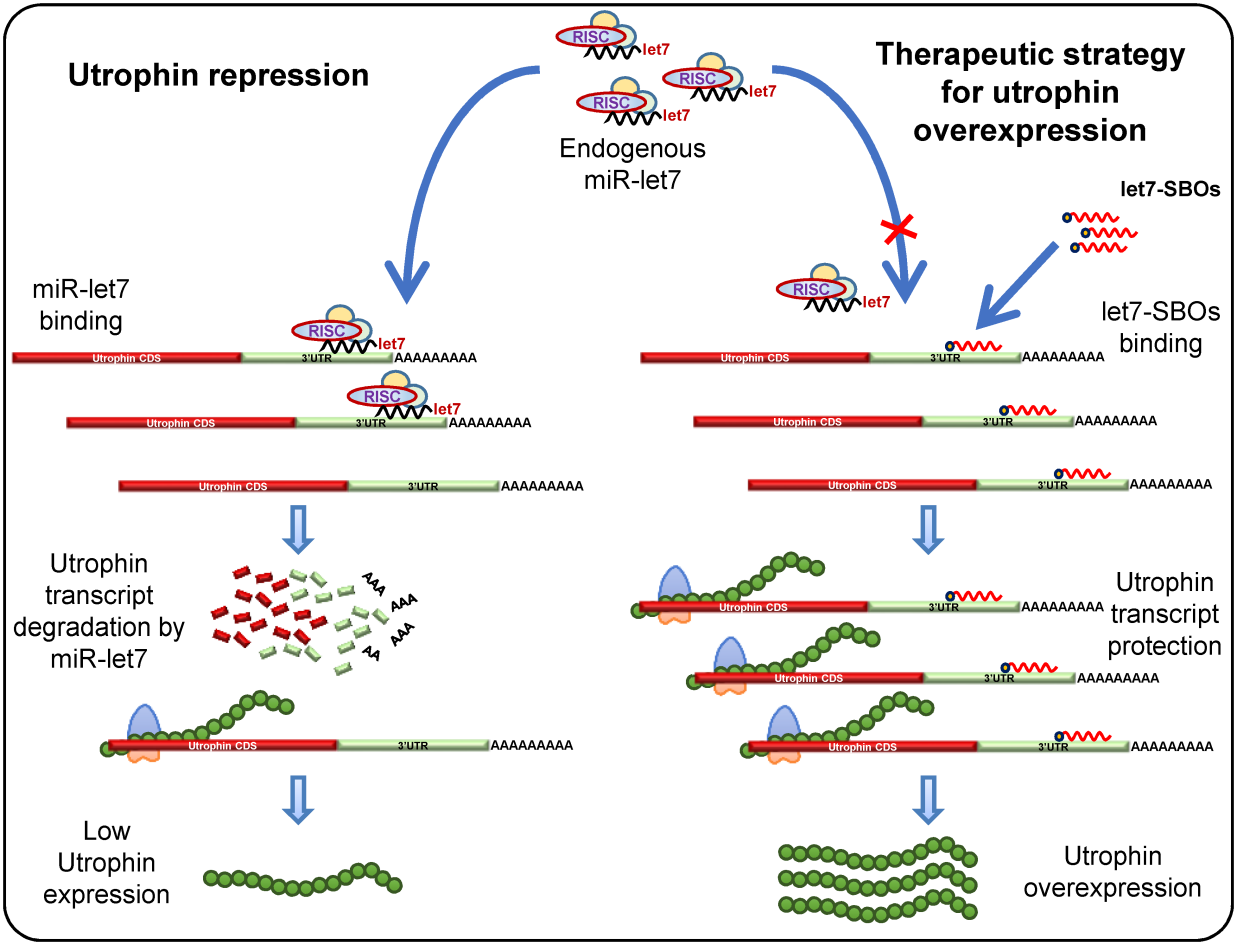
Therapeutic strategy for utrophin upregulation. Schematic representation for let-7 miRNA mediated repression of utrophin in muscle and therapeutic strategy for utrophin upregulation by let7-SBOs. In left panel, the 3’UTR of utrophin-A contains a let-7 binding site that is targeted by the let-7 miRNA, associated with a RNA-induced silencing complex (RISC) leading to translational repression and decreased levels of utrophin protein. Right Panel shows let7-SBOs block the endogenous let-7 binding site of 3’UTR, preventing miRNAs from binding. In this case, the normally occurring miRNA-mediated repression of utrophin will be repressed, leading to an increase in utrophin expression.

To develop and test this strategy, we designed and used let-7 site blocking oligonucleotides (SBOs) consisting of 2'-O-methyl modified bases on a phosphorothioate backbone that anneal to the utrophin 3’UTR at the let-7 family target site and prevent utrophin translational repression by let-7c or other let-7 family members [26]. These oligonucleotides have a high affinity for hybridization and do not induce RNase H-dependent cleavage [27]. They have been studied extensively in recent years as antisense therapeutic agents, which are protected against extra- and intracellular degradation by their modified structure. Synthetic oligonucleotides are being developed as therapies for a broad range of diseases, including hypertriglyceridemia [28], viral infections [29] and to induce dystrophin exon skipping for DMD [30–33]. Since let7-SBOs anneal to the utrophin 3’UTR, our blocking strategy is comparatively specific for utrophin (Fig 1), rather than affecting other let-7 target genes [34–36] as would be the case in using a let-7 miRNA sponge/antagomir strategy [37].

## Materials and methods

### Cell culture

The mouse muscle C2C12 myoblasts and human kidney HEK293T cell lines (both from ATCC) were cultured in DMEM with 10% FBS, glutamine, penicillin and streptomycin.

### Oligonucleotides

The let7-SBOs (5’-CUG AGG UAG AAA GGU GAU CAU GGC UC-3’) and control oligonucleotides (5’-GUG AGC ACU UCU UUC CUU CUU UUU U-3’), are 2′-O-methyl phosphorothioate RNA oligos with a phosphorothioate backbone. The let7-SBOs was designed to specifically target the let7 miRNA binding site in the utrophin 3’UTR. These oligonucleotides were synthesized on an Expedite 8909 Nucleic acid synthesizer using the 1 μmol thioate synthesis protocol supplied by the manufacturer.

### Constructs

For luciferase assays in stable cell lines, we used the previously described C2C12-5'3' cell line containing the 5'- and 3'-UTRs of utrophin flanking the luciferase coding sequence and stably expressed in mouse C2C12 cells [38].

For luciferase assays in human HEK293T cells, we generated the pGL4:50–5′Luc3′Hu construct by amplifying the 5′- (forward 5’-gtccaagcctGTATTGATGTCAAGCTGAACCA-3’ and reverse 5’-acttaagcctCTTGCCAGAGTTTCAAGATAATC-3’ primers) and 3′-UTRs (forward 5’-caggggccggccAGTATTCATCCGGCCAACC-3’ and reverse 5’-caaaggccggccGTGTTAAAATTACTTTTATTCAGGATG-3’ primers) of human utrophin and cloning them into the *Hind* III and *Fse* I sites that flank the luciferase coding sequence in the pGL4:50 vector (Promega, Madison, WI).

For transient transfections in mouse C2C12 cells, we used the previously described pGL3-5′Luc3′ construct containing the mouse 5′- and 3′-UTRs of utrophin flanking the luciferase site [26]. The Q5 Site-Directed Mutagenesis Kit (New England Biolabs) was used for deleting the let-7 site (24 bp sequence 5’-AGCCATGATCACCTTTCTACCTCA-3’; deletion of bases from 3’UTR of utrophin (Accession number: NM_007124.2) from pGL3-5′Luc3′ to create the pGL3-5′Luc3′-Δlet7 construct.

### Transfection

All oligonucleotide transfections were done using Lipofectamine RNAiMAX Transfection Reagent (Invitrogen) according to the manufacturer’s instructions. For oligonucleotide transfections, a 3: 1 ratio of Lipofectamine RNAiMAX (μl): μg oligonucleotides was used. For the transfection of plasmid constructs we used the LF3000 Transfection Reagent (Invitrogen) according to the manufacturer’s instructions.

### Luciferase reporter assays

Cells (C2C12 or HEK293T) were plated in 24 well plates at 30,000 cells per well, 1 day before transfection. 500 ng pGL3-5’Luc3’, pGL3-5'Luc3'-Δlet7 or equimolar amounts of other constructs were transfected, with 50 ng pRL-TK (Promega) and 100 nM let7-SBOs or control oligonucleotides, per well. Reporter activity was measured using the Dual Luciferase Assay (Promega) 24 hrs after transfection instructions using a TD 20/20 luminometer (Turner Designs, Sunnyvale, CA).

### Treatment of *mdx* mice with oligonucleotides and sample collection

Male *mdx* (C57BL/10ScSn-*Dmd*^*mdx*^/J) mice were obtained from The Jackson Laboratory (Bar Harbor, ME, USA). Mice were housed at the animal facility at the University of Pennsylvania before initiation of experiments. All experiments were approved by the Institutional Animal Care and Use Committee at the University of Pennsylvania.

For an *in vivo* proof-of-principle, a single dose of 20 μg let7-SBOs and unrelated control oligonucleotides was injected in tibialis anterior (TA) muscles of 1 month old male *mdx* mice (*n* = 3 for each group).

For systemic *in vivo* study the block randomization method was used to randomize mice into groups that result in equal sample sizes. Starting at an age of 1 month, *mdx* mice were treated intraperitoneally with low (10 mg; *n* = 3) and high (100 mg; *n* = 3) of let7-SBOs per kg body weight in 250 μl saline twice weekly for 1 month. For control 3 *mdx* mice in each group were injected intraperitoneally with low (10 mg) and high (100 mg) of control oligonucleotides per kg body weight in 250 μl saline twice weekly for 1 month.

Mice were sacrificed by carbon dioxide (CO_2_) euthanasia followed by cervical dislocation after the final injection. Blood samples were taken by cardiac puncture under deep terminal anesthesia for serum analysis. Serum was collected by centrifuging at 2,000 *g* for 5 min and it was stored at -80°C until analysis. After sacrifice, muscles and tissues were isolated, embedded in OCT and frozen in liquid nitrogen-cooled Isopentane, and stored at -80°C. Investigators were not blinded for the study. For all experiments sample sizes (*n*) are indicated in each figure legend.

### *Ex vivo* physiological assessment of skeletal muscle

Physiological properties, including isometric twitch force, isometric tetanic force, and force drop after ECCs, were quantified on freshly isolated EDL muscles from 2 months old *mdx* mice using an Aurora Mouse 1200A System equipped with Dynamic Muscle Control v.5.3 software, as described previously [39, 40]. EDL muscles were maintained in constantly oxygenated Ringer's solution (100 mM NaCl, 4.7 mM KCl, 3.4 mM CaCl_2_, 1.2 mM KH_2_PO_4_, 1.2 mM MgSO_4_, 25 mM HEPES and 5.5 mM D-glucose) at 24°C. The twitch stimulation protocol applied was a single stimulus with a duration of 0.2 ms. For measuring tetanic maximal force generation, the stimulus was repeated at a frequency of 120 Hz for 500 ms. Five min were allowed between two tetanic contractions to ensure muscle recovery. Muscle length was adjusted to obtain the maximal twitch response and this length was measured and recorded as optimal length (L_0_). Muscle cross-sectional area (CSA) of EDL muscles was calculated by dividing the muscle mass by the product of the muscle density coefficient (1.06 g/cm^3^), muscle L_0_, and the fiber length coefficient (0.45 for EDL). Specific force was determined by normalizing maximum isometric tetanic force to CSA.

After testing the isometric properties of EDL, a series of five ECCs was applied. The force drop was calculated as the percent difference in tetanic force between the first and fifth ECC. At the end of the physiological assessment, EDL muscles were embedded in OCT and frozen in liquid nitrogen-cooled Isopentane, and stored at -80°C.

### Western blotting

Western blotting was performed as described [26]. Cells and mouse muscles were processed in TNEC lysis buffer (1.5 mM Tris-HCl pH 8, 2.15 mM NaCl, 3.1% IGEPAL CA-630, 4.2 mM EDTA with Complete Protease Inhibitors-Roche). Protein concentration was assayed using a BCA Protein Assay Kit (Pierce). Approximately 30–40 μg protein were denatured with LDS sample buffer and NuPAGE reducing reagent (both Invitrogen) and heated to 72°C in for 10 min, then separated on 3–8% Tris-Acetate gels (Invitrogen) with Tris-Acetate running buffer for 1.5 hrs at 100 V. Proteins were transferred to nitrocellulose membranes for 15 min at 25 V in ice-cold transfer buffer (25 mM Tris-Cl pH 8.3, 192 mM glycine, 20% methanol, 0.05% sodium dodecyl sulfate) using Trans-Blot Turbo transfer system (BioRad). Efficiency of transfer and the even loading of lanes was verified by using post-transfer Ponceau-S staining of the membrane. After digital scanning Ponceau-S staining was removed by TBST washing. Membranes were blocked for 1 hr at room temperature in 5% non-fat milk in TBST (50 mM Tris-Cl pH 7.5, 150 mM NaCl, 0.1% Tween 20), then probed for utrophin (upper half of membrane) with mouse monoclonal anti-utrophin antibody MANCHO3 clone 8A4 (developed by Glenn E. Morris and obtained from the Developmental Studies Hybridoma Bank, Iowa) diluted 1:50 in 0.5% non-fat milk in TBST, or α-tubulin (lower half of membrane) with anti-α-tubulin antibody clone DM1A (Sigma) or vinculin with anti-vinculin (7F9) mouse antibody (Santa Cruz Biotechnology) diluted 1:2500 in 0.5% non-fat milk in TBST, for 1 hr at room temperature. For probing c-Myc, Stat3 and Jak3 proteins anti-c-Myc Rabbit (D3N8F) mAb (1:1000 dilution), anti-Stat3 (D3Z2G) Rabbit mAb (1:1000 dilution) and anti-Jak3 Rabbit mAb (1:1000 dilution) were used in 0.5% non-fat milk in TBST, for 1 hr at room temperature. These antibodies were obtained from Cell Signaling Technology, Inc. Membranes were washed in 3 changes of TBST for 5 min each, then incubated with HRP-conjugated goat-anti-mouse IgG (Santa Cruz Biotechnology) or HRP-conjugated goat-anti-rabbit IgG (Santa Cruz Biotechnology), diluted 1:2500 in 0.5% non-fat milk in TBST (for utrophin or α-tubulin), for 1 hr at room temperature. TBST washes were repeated 3 times, then bands were visualized using SuperSignal West Pico Chemiluminescent Substrate (Thermo Fisher Scientific) and images obtained using G:Box chemiluminescence system (Syngene). For presentation clarity, images were then inverted to give dark bands on a light background. Band densities were quantified using AlphaEaseFC (Alpha Innotech Corp.).

### RNA isolation, reverse-transcription and quantitative real-time PCR analysis

Trizol reagent (Life Technologies) was used for total RNA isolation from mouse tissues (e.g., diaphragm, gastrocnemius and TA). 1 μg total RNA was converted to cDNA using random primers and SuperScript III First-Strand Synthesis System (Invitrogen). Quantitative PCR (*q*PCR) was performed on QuantStudio3 Real-Time PCR System (Applied Biosystems) using Power SYBR Green Master Mix (Applied Biosystems) and primers 5’-GCGTGCAGTGGACCATTTTTCAGATTTA-3’ and 5’-GCGTGCAGATCGAGCGTTTATCCATTTG-3’ for utrophin or 5’-GGGCATCACCACGAAAATCTC-3’ and 5’-CTGCCGTTGTCAAACACCT-3’ for RPLP0. Data was analyzed on QuantStudio Design & Analysis Software (Applied Biosystems). Expression levels of Utrophin mRNAs were normalized to the endogenous control RPLP0 using ΔΔCt method.

### Immunofluorescence analysis

Immunofluorescence staining of utrophin was performed on TA cryosections. Frozen sections (10μm thick) were blocked for 1 hr in PBS containing 3% BSA and 0.05% Triton-X100, followed by 1 hr incubation with specific primary antibody rabbit anti-utrophin polyclonal antibody (1:200) (C-19 sc-7459; Santa Cruz Biotechnology, Inc.) in PBS containing 2% goat serum. After three PBS washes, sections were incubated for 1 hr with secondary antibody. For secondary staining, goat Alexa-594 anti-rabbit (1:1000) (R37117; Molecular Probes, Inc.) with α-Bungarotoxin (α-BTX), Alexa Fluor 488 conjugate (1:500) (B-13422; Molecular Probes, Inc.) were used. Control tissue sections were processed simultaneously in the same manner. Slides were rinsed three times for 5 min in PBS and mounted in ProLong Gold Antifade Mountant (P36930; Molecular Probes, Inc.). The fluorescence digital images were acquired using an Olympus BX51 microscope at an objective magnification of x20 and Olympus DP12 digital camera.

### Muscle histology and morphology

Frozen muscle 10 μm sections were cut at the mid belly of TA and diaphragm. Sections were fixed in ice-cold methanol for 5 min and then processed for histological examination by H&E staining. The entire muscle section was imaged and analyzed. The single-fiber area distributions and total number of fibers were determined for each muscle from digital images acquired using an Olympus BX51 microscope at an objective magnification of x10 and Olympus DP12 digital camera and software. Morphometric measurements (i.e., centrally nucleated fiber, single-fiber minimal Feret’s diameter) were made using the ImageJ image-processing software (rsbweb.nih.gov/ij). Minimal Feret’s diameter values for each muscle section were plotted as a frequency histogram. Calculation of variance coefficients of the minimal Feret’s diameter was calculated as described by Briguet *et*. *al* [41].

### Serum CK quantification

Fresh, un-hemolysed serum was isolated from blood samples. Serum CK was measured with the indirect colorimetric Creatine Kinase-SL Assay kit (Genzyme Diagnostics P.E.I. Inc., Charlottetown, Canada) according to the manufacturer’s instructions.

### Hydroxyproline content

The content of the amino acid hydroxyproline has been used as a measure of the extent of fibrosis in dystrophic skeletal muscle. Hydroxyproline assay was performed as described [42]. The TA muscle and the diaphragm were used for hydroxyproline quantification assay.

### Statistical analysis

Data were analyzed using the GraphPad Prism v5 statistical software package (GraphPad Software, La Jolla, CA). Data are reported as means ± SD. F-test was performed to test equality of variance between populations / groups. For statistical significance Mann-Whitney *U* test, the 2-way analysis of variance (ANOVA) with a Bonferroni correction or Tukey’s multiple comparison tests with statistical significance set at *P* ≤ 0.05 was performed. Appropriate statistical tests have been mentioned in figure legends.

## Results

**Fig 2:**
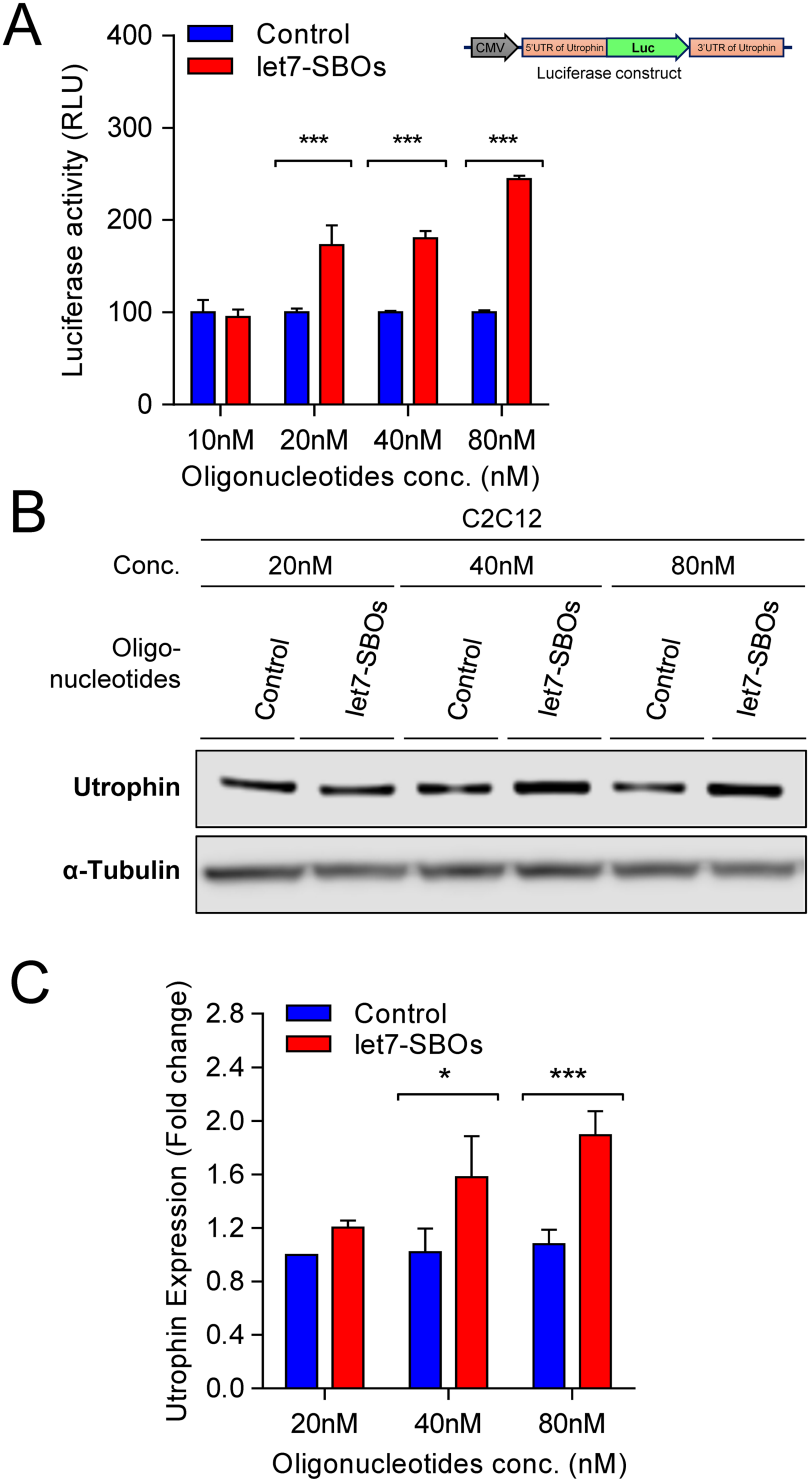
Therapeutic strategy for utrophin upregulation validation in C2C12 cells. (**A**) Efficacy of let7-SBOs in C2C12-5’Luc3’ utrophin reporter cells (cell line contains construct pGL4:50–5'Luc3' where reporter luciferase2 gene is flanked by the 5’- and 3’-UTRs of mouse utrophin-A). Cells transiently transfected with let7-SBOs /control oligonucleotides and luciferase activity measured 24 hrs post-transfection. Results shows significant increase in luciferase activity in C2C12-5’Luc3’ utrophin reporter cells 24 hrs of post-transfection with let7-SBOs compared to control oligonucleotides at various concentrations. Bars represent mean ± SD from 3 independent experiments. Statistical analysis was performed by 2-way ANOVA for multiple comparison followed by Bonferroni correction (***P ≤ 0.001). (**B**) Endogenous utrophin protein expression in C2C12 cells 24 hrs after transfection with let7-SBOs or control oligonucleotides at various concentrations, assayed by western blotting. α-Tubulin staining was used to control for equal loading. (**C**) Quantification of utrophin normalized to α-tubulin band density in western blot assay. Bars represent mean ± SD from 3 independent experiments and control. Control oligonucleotides treatment used as reference for utrophin expression in each independent experiment. Statistical analysis was performed by 2-way ANOVA for multiple comparison followed by Bonferroni correction (*P ≤ 0.05, ***P ≤ 0.001).

To validate the blocking strategy, let7-SBOs was transiently transfected into mouse C2C12-5’Luc3’ utrophin reporter cells, obtaining increased luciferase activity in a dose dependent manner (Fig 2A). Increased endogenous utrophin expression was also noted in C2C12 cells after let7-SBOs transfection at different concentrations (Fig 2B and 2C). The let7-SBOs treatment in human HEK293T cells also showed increase in luciferase activity as well as utrophin expression, demonstrating the applicability of this approach across species (S1 Fig). We also validated the specificity of let7-SBOs and requirement of the let-7c site in the utrophin 3’UTR for increasing utrophin expression. We developed a reporter construct (pGL3-5'Luc3'- Δlet7) in which the let-7c binding site in the utrophin 3’UTR region was deleted using site directed mutagenesis. Luciferase assays after 24 hrs of transient transfection of pGL3-5'Luc3'-Δlet7 construct with control and let7-SBOs, in C2C12 cells showed no difference in luciferase activity compared to upregulation noted using the pGL3-5'Luc3' construct (S2 Fig). For an *in vivo* proof-of-principle, a single dose of 20 μg let7-SBOs and unrelated control oligonucleotides was injected in tibialis anterior (TA) muscles of 1 month old male *mdx* mice. After 1 month, we observed c.a. 1.9-fold utrophin overexpression (S3 Fig).

**Fig 3:**
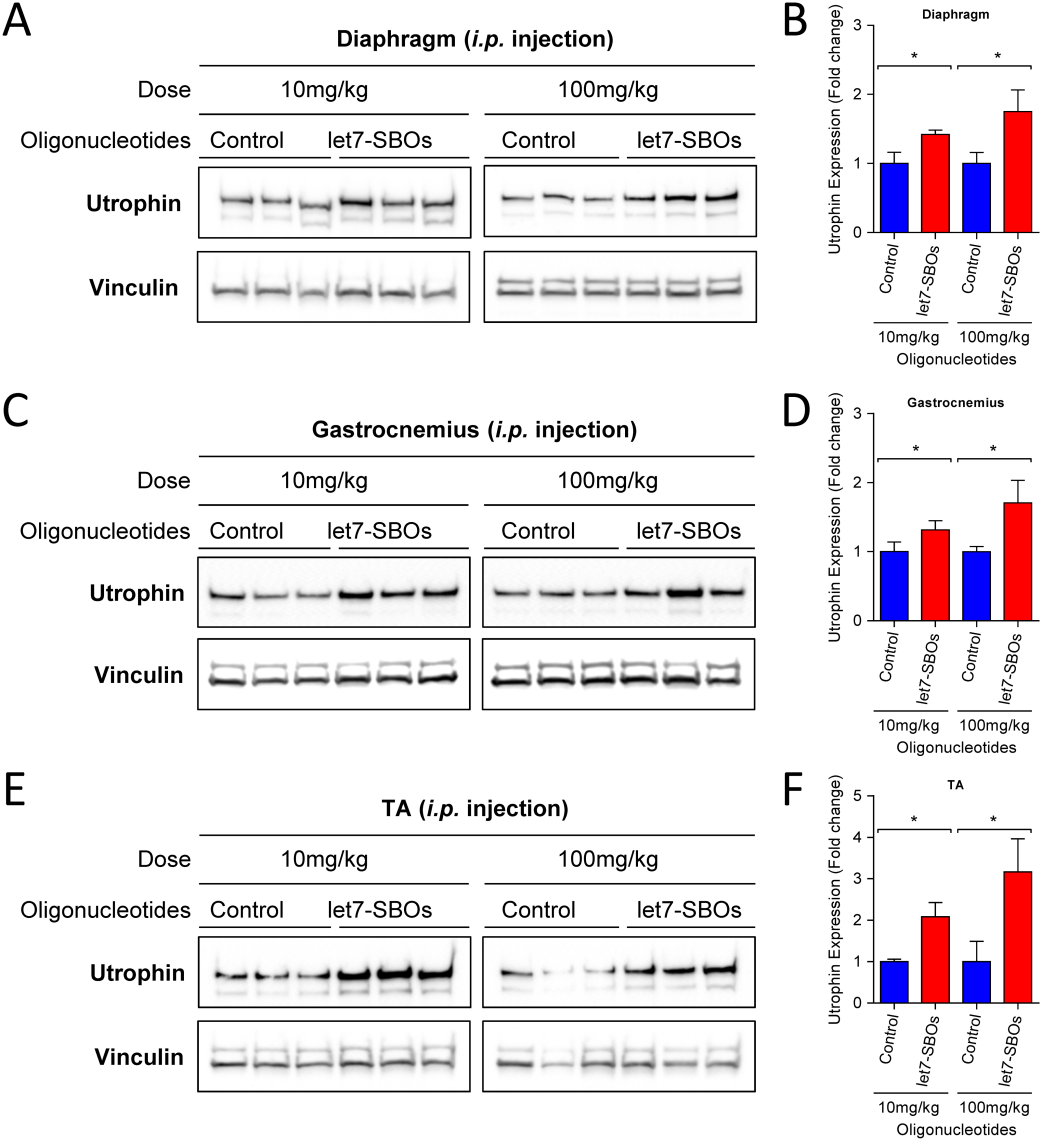
Effect of intraperitoneal let7-SBOs treatment on utrophin upregulation in muscles of 2 months old *mdx* mice after 1 month of treatment. Western blots and quantification of utrophin expression in diaphragm (**A**, **B**), gastrocnemius (**C, D**) and TA (**E, F**) muscles with low and high dose let7-SBOs treatment compared with control oligonucleotides. Vinculin was used to control for equal loading. Bands were densitometrically evaluated, normalized to Vinculin. Bars represent mean ± SD (*n* = 3 per group). Differences between groups were analyzed by the Mann-Whitney *U* test (*P ≤ 0.05).

**Fig 4:**
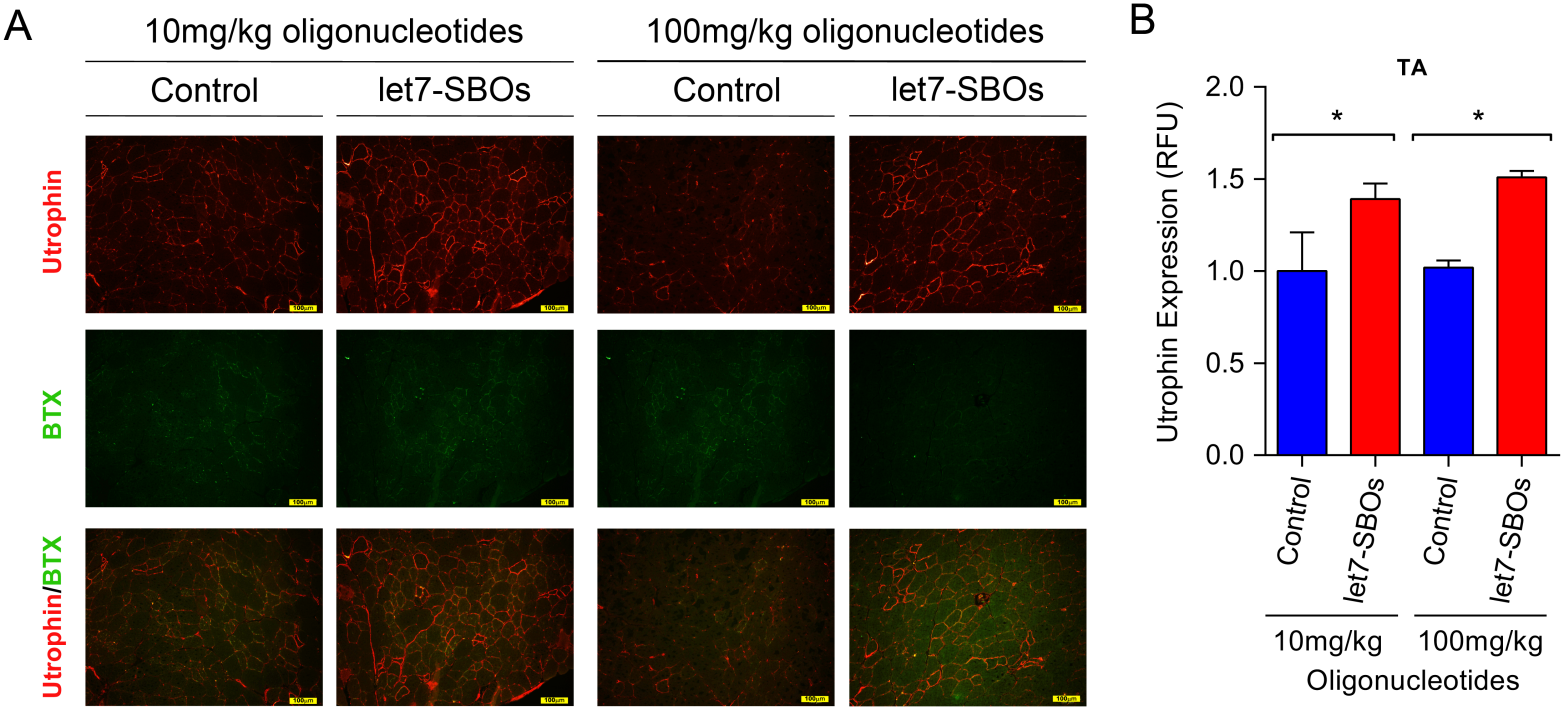
Utrophin expression in TA muscle of *mdx* mice treated with intraperitoneal injection of let7-SBOs. (**A**) Expression and localization of utrophin in *mdx* mice treated with let7-SBOs. Frozen sections of the TA muscles immuno-labelled with anti-utrophin antibodies and α-BTX. Utrophin labeling in regions of TA muscle with a paucity of neuromuscular junctions (demonstrated by lack of α-BTX staining). (Scale bar = 100 μm). (**B**) Relative fluorescence quantification of utrophin expression in TA muscles with low and high dose let7-SBOs treatment compared with control oligonucleotides. Frozen 10μm thick sections of the TA muscles immuno-labelled with utrophin antibodies. Bars represent mean ± SD (*n* = 3 mice per experimental group). Statistical comparison was analyzed by Mann-Whitney *U* test (*P ≤ 0.05).

**Fig 5:**
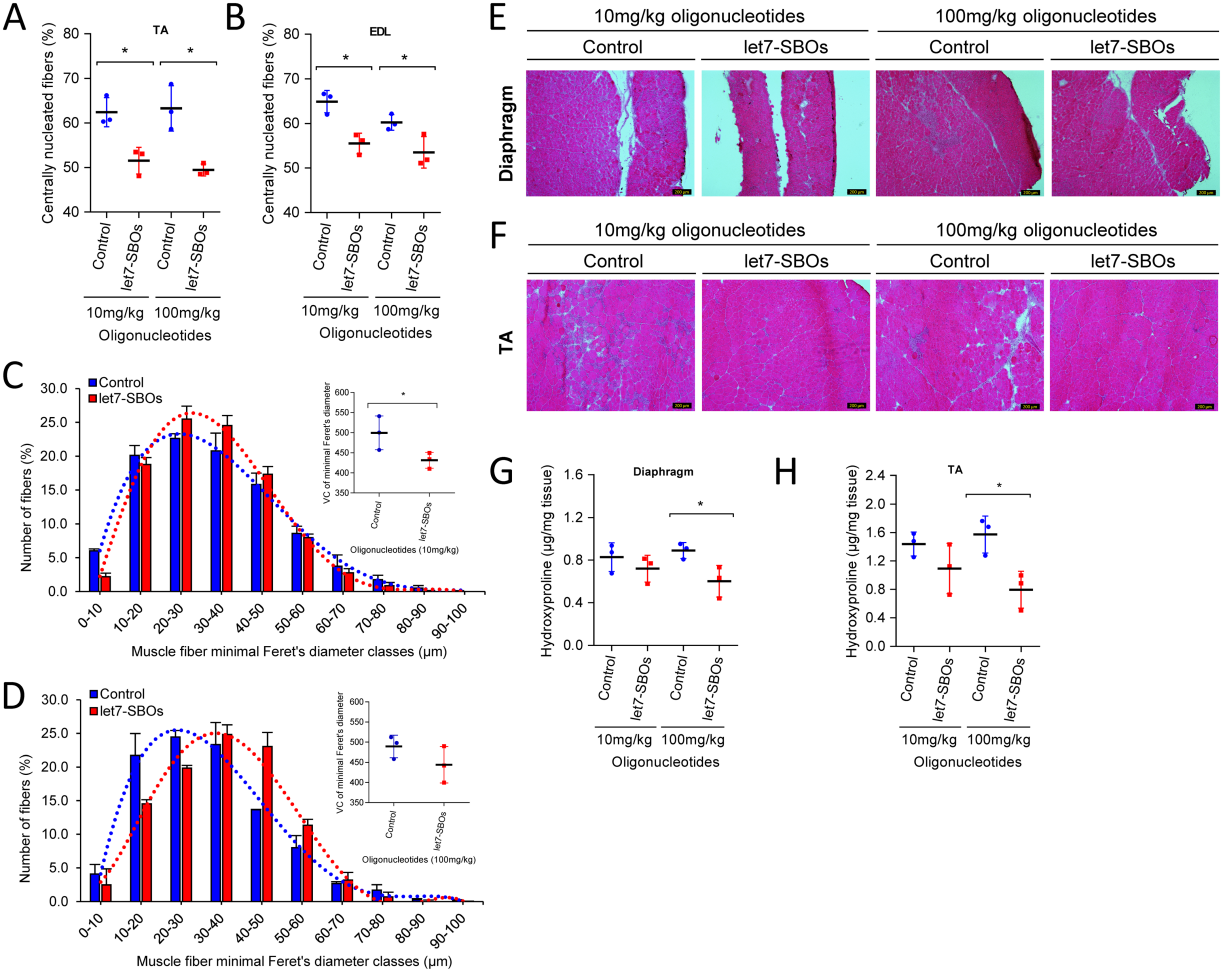
Effect of intraperitoneal let7-SBOs treatment on muscles morphology. Regeneration was quantified from the central nucleation counted from H&E-stained transverse TA (**A**) and EDL (**B**) muscles (*n* = 3 per group) sections from let7-SBOs treated *mdx* mice compared to the respective dose of control oligonucleotides (Mann-Whitney *U* test, *P≤0.05). Scatter dot plot represent mean ± SD (*n* = 3 per group). (**C, D**) Histogram distribution of EDL muscle fibers minimal Feret’s diameter in *mdx* mice (*n* = 3 per group) injected with low (**C**) and high (**D**) dose of let7-SBOs treated (red) and control oligonucleotides (blue). Variance coefficient of minimal Feret’s diameter are shown in inset graph (variance coefficient 500±24 versus 431 ± 11 in low dose group and 489 ± 16 versus 444 ± 26 in high dose group). Statistical analysis was performed by Mann-Whitney *U* test (*P ≤ 0.05). Data represent mean ± SD (*n* = 3 per group). (**E, F**) Representative images of H&E staining showing decreased muscle damage, lack of cellular infiltration and fibrosis resulting from low and high dose of let7-SBOs treatment in diaphragm (**E**) and TA (**F**) compared to the respective dose of Control oligonucleotides (Scale bar = 200 μm). (**G, H**) Muscles fibrosis analyzed by the hydroxyproline content of diaphragm (**G**) and TA (**H**) muscles from let7-SBOs treated *mdx* showing hydroxyproline content in high dose of let7-SBOs treatment compared to the respective dose of control oligonucleotides. Significant differences were assessed by Mann-Whitney *U* test (*P ≤ 0.05). Scatter dot plot represent mean ± SD (*n* = 3 per group).

To test the ability of let7-SBOs treatment to improve the dystrophic phenotype and obtain proof-of-concept *in vivo*, 1 month old male *mdx* mice were injected bi-weekly with intraperitoneal injection of let7-SBOs and control oligonucleotides at two different doses (low dose 10 mg/kg and high dose 100 mg/kg) for 1 month. After a month of treatment mice were sacrificed and analyzed by morphological, biochemical and physiological means. We observed c.a. 1.4- and 1.8-fold increase of utrophin expression in diaphragm (Fig 3A and 3B), 1.3 and 1.7-fold increase in the gastrocnemius muscle (Fig 3C and 3D) and 2.1- and 3.2-fold increase in the TA muscle (Fig 3E and 3F) of both low and high dose let7-SBOs treatment compared to control oligonucleotides groups by western blot analysis. We also measured level of utrophin mRNA expression in diaphragm, gastrocnemius and TA muscles (S4 Fig) and observed significant increase in utrophin mRNA expression in diaphragm (c.a. 1.3-fold) and TA muscles (c.a. 2.5-fold) of high dose let7-SBOs treatment. Utrophin is enriched at the neuromuscular junction and myotendinous junction in adult skeletal muscle cells and extends the entire sarcolemma in developing and regenerating muscle [10, 16]. Immunofluorescence labeling for utrophin showed c.a. 1.4 (low dose) and 1.5-fold (high dose) increased utrophin at both synaptic regions (S5 Fig) as well as in the extrasynaptic sarcolemma of fibers in the TA muscles of let7-SBOs treated *mdx* mice compared to controls (Fig 4A and 4B). Morphologically, dystrophic muscles typically show a higher percentage of centrally nucleated fibers (CNF's) [43, 44]. We observed significant reduction in the number of CNF's in TA (c.a. 11% and 14% reduction) and extensor digitorum longus (EDL) muscles (c.a. 9% and 7% reduction) in the low and high dose let7-SBOs treated mice, compared to controls (Fig 5A and 5B). Morphometric analyses of EDL muscles revealed a decrease in the variance coefficient of the minimal Feret’s diameter in the low dose regimen (Fig 5C and 5D), indicating a decrease fiber heterogeneity and suggesting an improvement in dystrophic pathology [41]. Details of these measurements and other morphometric parameters of measured are provided in Table 1. We next examined whether the dystrophic histopathology was improved by let7-SBOs treatment. Histological analysis showed a reduction in pathophysiological changes such as necrosis and cellular infiltration in diaphragm (Fig 5E) and TA (Fig 5F) muscles from treated *mdx* mice compared to controls in both low and high dose regimens. To determine whether the improvement in morphology was associated with biochemical improvement, we analyzed the hydroxyproline content of muscles as a biochemical marker for fibrosis [45]. A significant reduction of hydroxyproline was found in the diaphragm (Fig 5G) and TAs (Fig 5H) of *mdx* mice treated with high dose of let7-SBOs compared to controls. No significant decrease in serum creatine kinase (CK) was noted (S6 Fig). To quantify functional improvement, we analyzed physiological properties of EDL muscle (Table 1). EDL muscles from high dose let7-SBOs treated mice showed increased specific strength compared to controls (Table 1). No changes were noted in post eccentric lengthening contraction (ECC) force drop (S7 Fig). Finally, we performed western assay for other let-7 target genes (e.g. c-Myc [34], Stat3 [35], Jak3 [36] etc.) to test our blocking strategy is comparatively specific for utrophin, and we have not observed any change in expression of these let-7 target genes (S8 Fig).

**Table 1:** Comparison of morphological and physiological properties of EDL muscle.

	10mg/kg dose		100mg/kg dose	
	Control oligonucleotides	let7-SBOs	Control oligonucleotides	let7-SBOs
Weight (mg)	14.5 ± 1.0 (3)	14.5 ± 2.4 (3)	15.4 ± 0.5 (3)	12.2 ± 0.6** (3)
CSA (mm^2^)	2.5 ± 0.2 (3)	2.4 ± 0.4 (3)	2.5 ± 0.1 (3)	2.1 ± 0.1** (3)
Absolute force (mN)	352.5 ± 56.7 (3)	382.4 ± 81.6 (3)	395.5 ± 13.6 (3)	392.8 ± 17.3 (3)
Specific force (N cm^-2^)	14.0 ± 2.8 (3)	15.8 ± 2.2 (3)	15.8 ± 1.2 (3)	18.7 ± 0.6* (3)
ECC force decrease (1–5) (%)	36.3 ± 5.7 (3)	40.2 ± 0.4 (3)	51.5 ± 18.0 (3)	44.1 ± 10.7 (3)
ECC force drop (5^th^) (%)	63.7 ± 5.7 (3)	59.8 ± 0.4 (3)	48.5 ± 18.0 (3)	55.9 ± 10.7 (3)
Avg. of Minimal Feret’s diameter (μM)	32.01 ± 15.9 (1521)	32.24 ±13.8 (2046)	30.6 ± 15.4 (1174)	35.5 ± 16.2*** (1232)
Variance coefficient of Min. Feret’s diameter	499.5 ± 24.2 (3)	431.3 ± 11.4* (3)	489.4 ± 16.1 (3)	444.0 ± 26.1 (3)

Results are represented as mean ±SD; numbers in parentheses are *n*; asterisks, each dose of let7-SBOs treatment group compared with respective dose of control oligonucleotides and statistical significance was analyzed by Mann-Whitney *U* test

(*P≤0.05,

**P ≤ 0.01,

***P ≤ 0.001).

CSA, cross-sectional area.

## Discussion

miRNAs play an important role in the post-transcriptional control of utrophin expression. Rosenberg et al, (2006) showed that the miR-206 targets Utrophin [25]. We had previously found five additional miRNAs namely, let-7c, miR-150, miR-196b, miR-296-5p, miR-133b could repress utrophin expression [26]. We chose let-7c as an appropriate initial candidate to test our strategy since it is highly expressed in fast and slow skeletal muscles, and its antisense inhibition in C2C12 cells caused a translational upregulation of the luciferase reporter [26]. By utilizing let7-SBOs targeting 3’UTR instead of let-7c miRNA itself, biological functions of let-7 miRNA other than utrophin regulation, should remain unaffected, thus increasing specificity. Our data presented in this study demonstrate a novel therapeutic strategy for DMD based on inhibiting the utrophin:let-7c miRNA interaction using let7-SBOs. Systemic treatment of *mdx* mice using let7-SBOs, led to utrophin upregulation and functional improvement of the dystrophic phenotype. While only two doses were used in this study, there was in general, a good correlation between oligomer dose and response. The higher dose resulted in greater utrophin expression and improvement compared to low dose treatment. However, even the high dose treatment did not completely improve dystrophic changes; serum CK and susceptibility to damage by ECCs were not improved. This might be related to initiating let7-SBOs treatment at 1 month of age rather than in the prenatal period [46], an inadequate dosage, an inadequate duration of treatment or an inherent limitation of the strategy itself. Additional modifications or use of a different platform chemistry may increase the ultimate pharmacological effectiveness of the let7-SBO approach such as has been noted with morpholinos (PMOs) [47]. Given the variability to therapeutic response noted across different muscles as well as in short vs long terms studies [48, 49], additional studies would be needed in different animal models to fully determine the limitations of the present strategy. It is noteworthy that delivery of let7-SBOs by simple intraperitoneal injections circumvents the need for using specialized viral vectors to deliver genes and the potential problems with toxicity or immune response against the vector or recombinant dystrophin molecule itself. Additionally, our approach could also be used in combination with dystrophin or non-dystrophin-based therapies for DMD and may help potentiate these approaches as well.

## Supporting information

S1 Fig Efficacy of let7-SBOs in human HEK293T cells. (**A**) HEK293 cells transiently transfected with firefly luciferase reporter construct pGL4:50–5'Luc3'Hu (the reporter luciferase2 gene is flanked by the 5’- and 3’-UTRs of human utrophin-A) and let7-SBOs / control oligonucleotides. Figure shows luciferase activity in HEK293T cells 24 hrs after transfection with let7-SBOs compared to control oligonucleotides at various concentrations. Bars represent mean ± SD from 3 independent experiments. Statistical analysis was performed by 2-way ANOVA for multiple comparison followed by Bonferroni correction, **P ≤ 0.01, ***P ≤ 0.001. (**B**) Endogenous utrophin protein expression in HEK293T cells after 24 hrs of transient transfection with let7-SBOs or control oligonucleotides at different concentrations was assayed by western blotting. (**C**) Quantification of utrophin band density normalized to α-tubulin band density in western blot assay. Bars represent mean ± SD from 3 independent experiments. Statistical analysis was performed by 2-way ANOVA for multiple comparison followed by Bonferroni correction (*P ≤ 0.05, ***P ≤ 0.001).
(PDF)

S2 Fig Luciferase activity of C2C12 cells transiently transfected with pGL3-5'Luc3', pGL3-5'Luc3'-Δlet7 construct and let7-SBOs. (**A**) Schematics of the WT reporter construct pGL3-5'Luc3' (luciferase reporter flanked by the 5’- and 3’-UTRs of mouse utrophin-A) and pGL3-5'Luc3'-**Δ**let7 reporter construct (luciferase reporter flanked by the 5’- and 3’-UTRs of mouse utrophin-A in which the let-7c binding site has been deleted) (**B**) C2C12 cells were transiently transfected with pGL3-5'Luc3' or pGL3-5'Luc3'-**Δ**let7 along with control oligonucleotides (blue) or let7-SBOs (red). Figure shows luciferase activity measured after 24 hrs of transfection. Bars represent mean ± SD from 4 independent experiments. Statistical analysis was performed by 2-way ANOVA for multiple comparison followed by Bonferroni correction (*P ≤ 0.01).
(PDF)

S3 Fig Utrophin expression in TA muscle of *mdx* mice treated with intramuscular injection of let7-SBOs. (**A**) Utrophin expression in TA muscles of *mdx* mice (*n* = 3 per group) with intramuscular injection of let7-SBOs and control oligonucleotides. α-Tubulin staining was used to control for equal loading. (**B**) Quantification of utrophin normalized to α-tubulin band density in western blot assay. Bars represent mean ± SD (*n* = 3 mice per experimental group). Statistical comparison was analyzed by Mann-Whitney *U* test (*P ≤ 0.05).
(PDF)

S4 Fig Transcriptional expression of utrophin in *mdx* mice treated with intraperitoneal injection of let7-SBOs. (**A-C**) Utrophin mRNA expression by RT-qPCR in diaphragm (**A**), gastrocnemius (**B**) and TA (**C**) muscles of *mdx* mice (*n* = 3 per group) with intramuscular injection of let7-SBOs and control oligonucleotides. *RPLP0* was used as housekeeping gene. Bars represent mean ± SD (*n* = 3 mice per experimental group). Statistical comparison was analyzed by Mann-Whitney *U* test (*P ≤ 0.05).
(PDF)

S5 Fig Utrophin expression in TA muscle of *mdx* mice treated with intraperitoneal injection of let7-SBOs. Expression and localization of utrophin in *mdx* mice treated with let7-SBOs. Frozen sections of the TA muscles immuno-labelled with anti-utrophin antibodies and α-BTX. Utrophin labeling in neuromuscular junction-rich regions (demonstrated by α-BTX staining) of TA muscle (Scale bar = 100 μm).
(TIFF)

S6 Fig Effect of let7-SBOs treatment in serum CK activity. Decrease in serum CK activity in *mdx* mice treated with the low dose (**A**) and high dose (**B**) of let7-SBOs compared to control oligonucleotides injected *mdx* mice. Scatter dot plot represent means ± SD (*n* = 3 in each group). Statistical analysis was performed by Mann-Whitney *U* test (*P ≤ 0.05) to low and high dose treatment group, respectively.
(PDF)

S7 Fig Comparisons of drop in ECC force after five successive ECC’s of EDL muscles of *mdx* mice. Force drop after five successive ECC’s in EDL muscles of *mdx* mice treated with low (**A**) and high (**B**) dose of let7-SBOs and control oligonucleotides (*n* = 3 for each group). Significant differences were assessed by 2-way ANOVA for multiple comparisons followed by Bonferroni correction (*P ≤ 0.05).
(PDF)

S8 Fig Effect of let7-SBOs on other let7 target genes. Western blots and quantification of other let-7 target genes c-Myc (**A, B**), Stat3 (**C, D**) and Jak3 (**E, F**) in gastrocnemius muscles with low and high dose let7-SBOs treatment compared with control oligonucleotides. Vinculin was used to control for equal loading. Bands were densitometrically evaluated, normalized to Vinculin. Significant differences were assessed by Mann-Whitney *U* test (*P ≤ 0.05). Bars represent mean ± SD (*n* = 3 per group).
(PDF)

S1 Table Body and muscle weight of *mdx* mice. (DOC)
