# Supplementary figures and images for: Functional improvement of dystrophic muscle by repression of utrophin: let-7c interaction
Source: PLoS One. 2017 Oct 18;12(10):e0182676. doi: 10.1371/journal.pone.0182676 (PMC5646768; doi:10.1371/journal.pone.0182676)

# S1 Figure

A

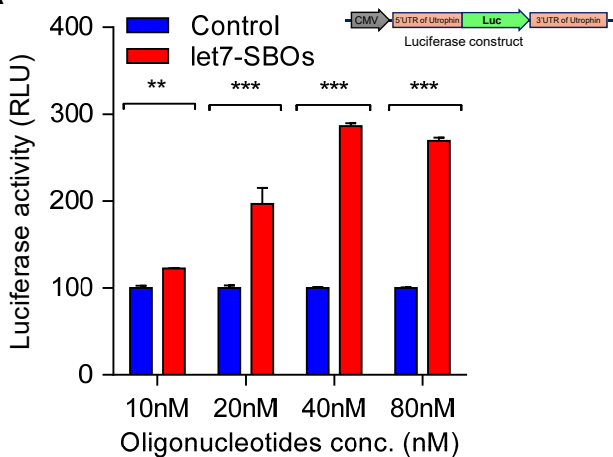

B

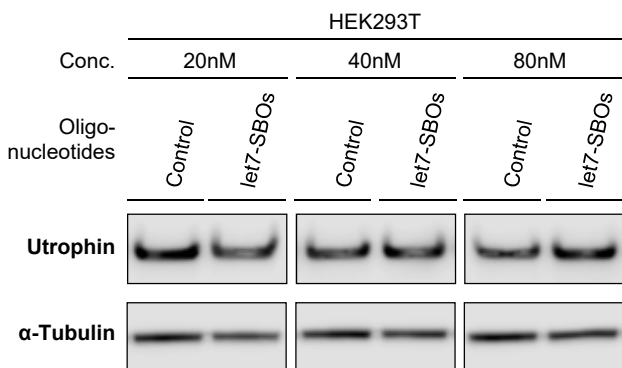

C

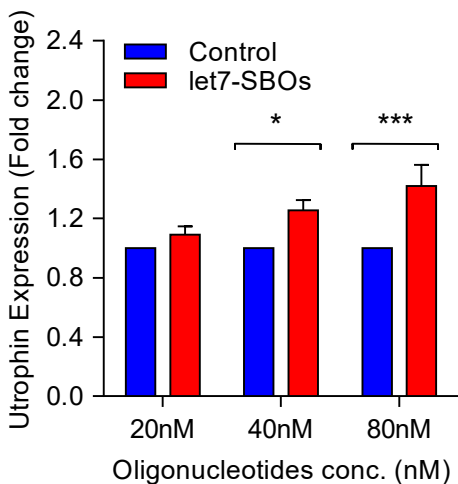

Supplement: S1 Fig — (A) HEK293 cells transiently transfected with firefly luciferase reporter construct pGL4:50–5'Luc3'Hu (the reporter luciferase2 gene is flanked by the 5’- and 3’-UTRs of human utrophin-A) and let7-SBOs / control oligonucleotides. Figure shows luciferase activity in HEK293T cells 24 hrs after transfection with let7-SBOs compared to control oligonucleotides at various concentrations. Bars represent mean ± SD from 3 independent experiments. Statistical analysis was performed by 2-way ANOVA for multiple comparison followed by Bonferroni correction, **P ≤ 0.01, ***P ≤ 0.001. (B) Endogenous utrophin protein expression in HEK293T cells after 24 hrs of transient transfection with let7-SBOs or control oligonucleotides at different concentrations was assayed by western blotting. (C) Quantification of utrophin band density normalized to α-tubulin band density in western blot assay. Bars represent mean ± SD from 3 independent experiments. Statistical analysis was performed by 2-way ANOVA for multiple comparison followed by Bonferroni correction (*P ≤ 0.05, ***P ≤ 0.001). (PDF) [file pone.0182676.s001.pdf]

# S2 Figure

A

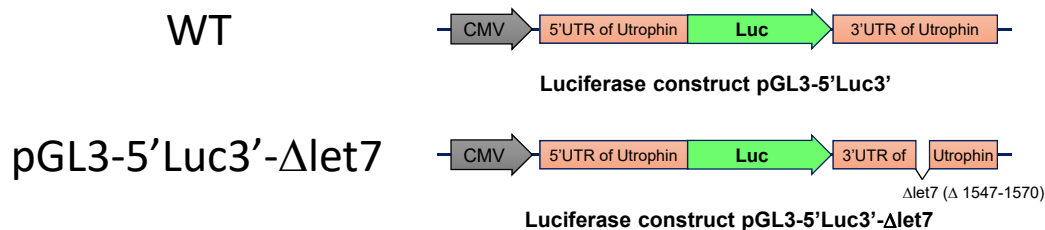

B

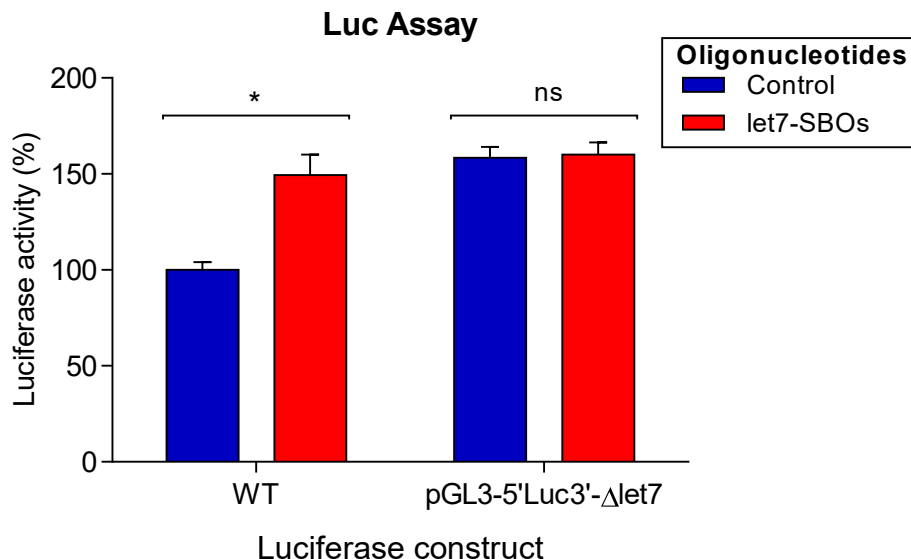

Supplement: S2 Fig — (A) Schematics of the WT reporter construct pGL3-5'Luc3' (luciferase reporter flanked by the 5’- and 3’-UTRs of mouse utrophin-A) and pGL3-5'Luc3'-Δlet7 reporter construct (luciferase reporter flanked by the 5’- and 3’-UTRs of mouse utrophin-A in which the let-7c binding site has been deleted) (B) C2C12 cells were transiently transfected with pGL3-5'Luc3' or pGL3-5'Luc3'-Δlet7 along with control oligonucleotides (blue) or let7-SBOs (red). Figure shows luciferase activity measured after 24 hrs of transfection. Bars represent mean ± SD from 4 independent experiments. Statistical analysis was performed by 2-way ANOVA for multiple comparison followed by Bonferroni correction (*P ≤ 0.01). (PDF) [file pone.0182676.s002.pdf]

# S3 Figure

## A

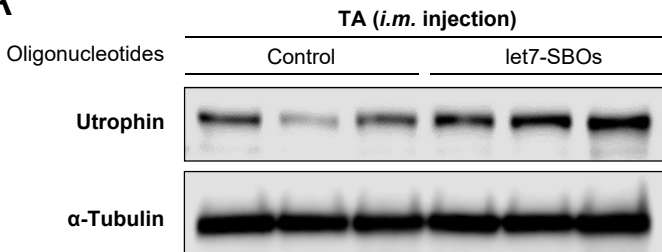

## B

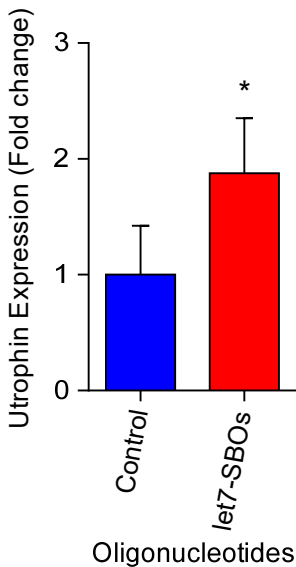

Supplement: S3 Fig — (A) Utrophin expression in TA muscles of mdx mice (n = 3 per group) with intramuscular injection of let7-SBOs and control oligonucleotides. α-Tubulin staining was used to control for equal loading. (B) Quantification of utrophin normalized to α-tubulin band density in western blot assay. Bars represent mean ± SD (n = 3 mice per experimental group). Statistical comparison was analyzed by Mann-Whitney U test (*P ≤ 0.05). (PDF) [file pone.0182676.s003.pdf]

# S4 Figure

A

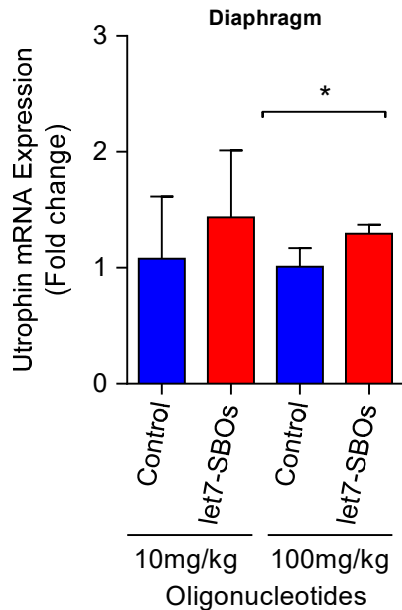

B

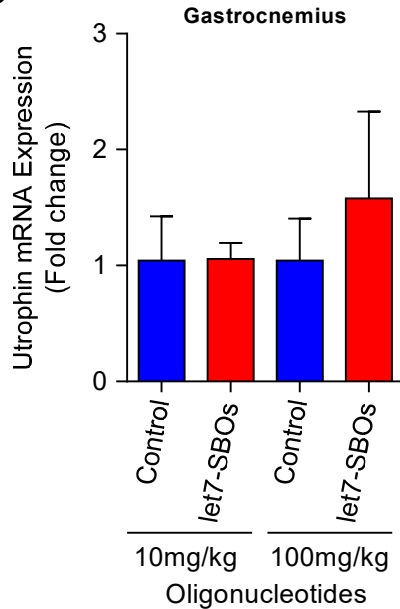

C

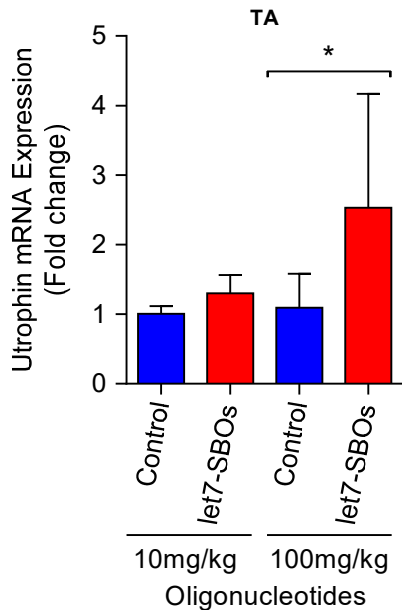

Supplement: S4 Fig — (A-C) Utrophin mRNA expression by RT-qPCR in diaphragm (A), gastrocnemius (B) and TA (C) muscles of mdx mice (n = 3 per group) with intramuscular injection of let7-SBOs and control oligonucleotides. RPLP0 was used as housekeeping gene. Bars represent mean ± SD (n = 3 mice per experimental group). Statistical comparison was analyzed by Mann-Whitney U test (*P ≤ 0.05). (PDF) [file pone.0182676.s004.pdf]

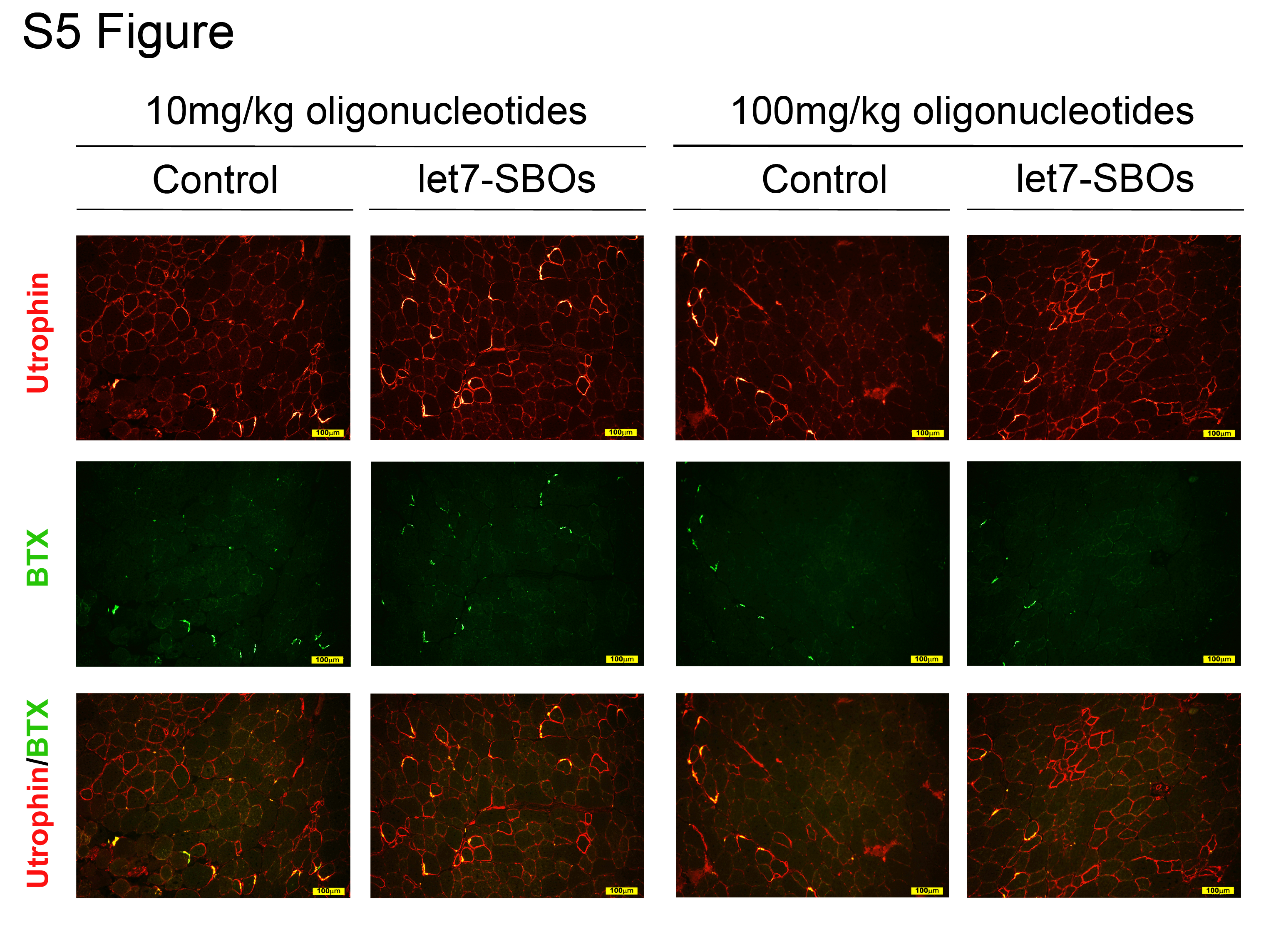

Supplement: S5 Fig — Expression and localization of utrophin in mdx mice treated with let7-SBOs. Frozen sections of the TA muscles immuno-labelled with anti-utrophin antibodies and α-BTX. Utrophin labeling in neuromuscular junction-rich regions (demonstrated by α-BTX staining) of TA muscle (Scale bar = 100 μm). (TIFF) [file pone.0182676.s005.tiff]

# S6 Figure

A

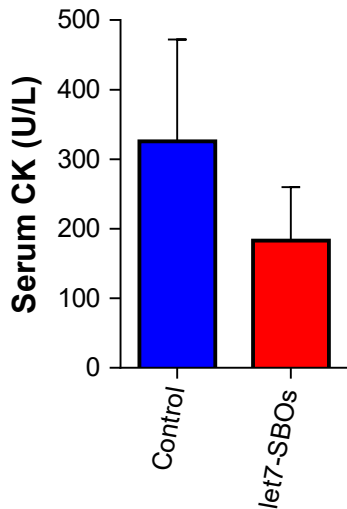

Oligonucleotides (10mg/kg)

B

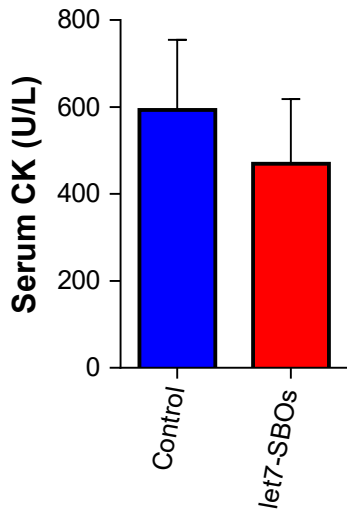

Oligonucleotides (100mg/kg)

Supplement: S6 Fig — Decrease in serum CK activity in mdx mice treated with the low dose (A) and high dose (B) of let7-SBOs compared to control oligonucleotides injected mdx mice. Scatter dot plot represent means ± SD (n = 3 in each group). Statistical analysis was performed by Mann-Whitney U test (*P ≤ 0.05) to low and high dose treatment group, respectively. (PDF) [file pone.0182676.s006.pdf]

# S7 Figure

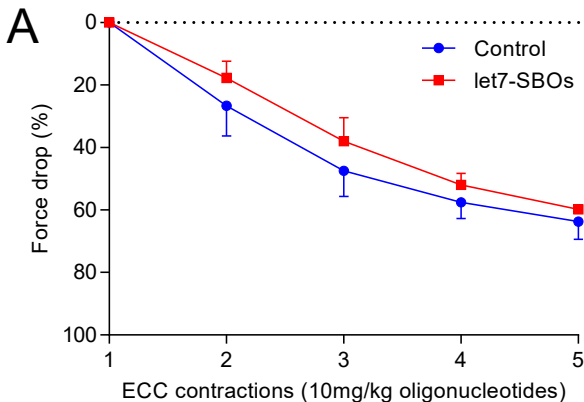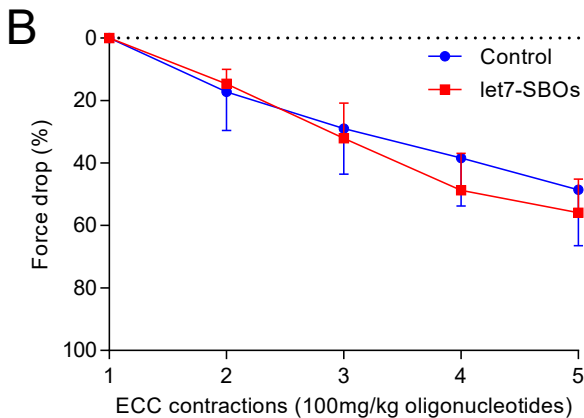

Supplement: S7 Fig — Force drop after five successive ECC’s in EDL muscles of mdx mice treated with low (A) and high (B) dose of let7-SBOs and control oligonucleotides (n = 3 for each group). Significant differences were assessed by 2-way ANOVA for multiple comparisons followed by Bonferroni correction (*P ≤ 0.05). (PDF) [file pone.0182676.s007.pdf]

# S8 Figure

## A

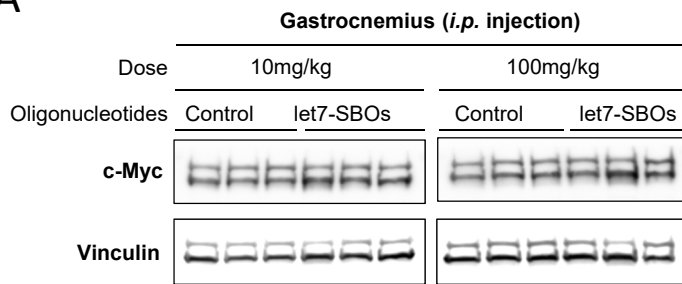

## B

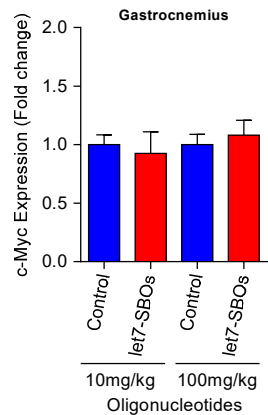

## C

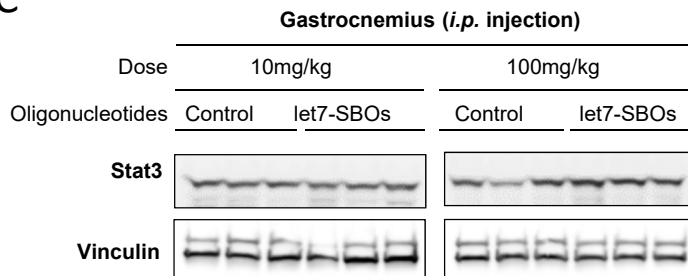

## D

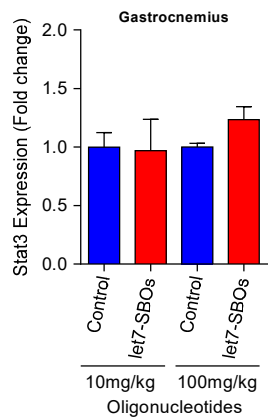

## E

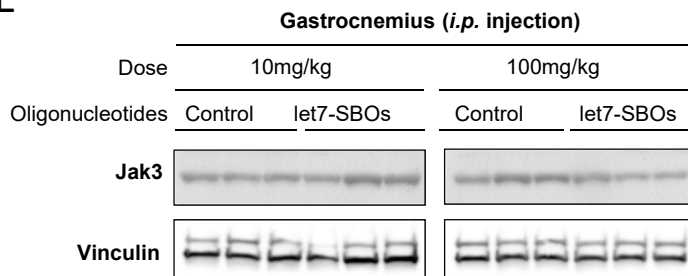

## F

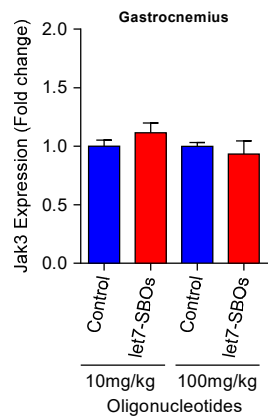

Supplement: S8 Fig — Western blots and quantification of other let-7 target genes c-Myc (A, B), Stat3 (C, D) and Jak3 (E, F) in gastrocnemius muscles with low and high dose let7-SBOs treatment compared with control oligonucleotides. Vinculin was used to control for equal loading. Bands were densitometrically evaluated, normalized to Vinculin. Significant differences were assessed by Mann-Whitney U test (*P ≤ 0.05). Bars represent mean ± SD (n = 3 per group). (PDF) [file pone.0182676.s008.pdf]
