# Supplementary material for: Functional improvement of dystrophic muscle by repression of utrophin: let-7c interaction
Source: PLoS One. 2017 Oct 18;12(10):e0182676. doi: 10.1371/journal.pone.0182676 (PMC5646768; doi:10.1371/journal.pone.0182676)
Supplement: S1 Table — (DOC) [file pone.0182676.s009.doc]

**S1 Table: Body and muscle weight of *mdx* mice**

|  | **10mg/kg dose** | | **100mg/kg dose** | |
| --- | --- | --- | --- | --- |
|  | Control oligonucleotides | let7-SBOs | Control oligonucleotides | let7-SBOs |
| Pre study body weight (g) | 22.2 ± 0.6 (3) | 22.4 ± 0.6 (3) | 23.2 ±0.4 (3) | 22.6 ± 0.4 (3) |
| Post study body weight (g) | 28.4 ± 0.3 (3) | 28.3 ± 1.9 (3) | 29.3 ± 2.1 (3) | 27.5 ± 0.7 (3) |
| TA weight (mg) | 70.0 ± 3.4 (3) | 71.0 ± 6.6 (3) | 67.9 ± 6.2 (3) | 60.6 ± 1.1 (3) |
| EDL weight (mg) | 14.5 ± 1.0 (3) | 14.5 ± 2.4 (3) | 15.4 ± 0.5 (3) | 12.2 ± 0.6** (3) |

Results are represented as means ± SD; numbers in parentheses are *n*; asterisks, each dose of let7-SBOs treatment group compared with respective dose of control oligonucleotides and statistical significance was analyzed by Mann-Whitney *U* test, (***P* ≤ 0.01).
